# DWI in the Differentiation of Malignant and Benign Breast Lesions Presenting with Non-Mass Enhancement on CE-MRI

**DOI:** 10.3390/cancers17010031

**Published:** 2024-12-25

**Authors:** Iva Perić, Boris Brkljačić, Tade Tadić, Kristian Jerković, Krešimir Dolić, Matija Borić, Marija Ćavar

**Affiliations:** 1 Department of Diagnostic and Interventional Radiology, University Hospital Split, Spinčićeva 1, 21000 Split, Croatia; ivaperic@kbsplit.hr (I.P.); ttadic@kbsplit.hr (T.T.); kjerkovic@kbsplit.hr (K.J.); kdolic@kbsplit.hr (K.D.); 2 Department of Diagnostic and Interventional Radiology, University Hospital “Dubrava”, University of Zagreb School of Medicine, Avenija Gojka Šuška 6, 10000 Zagreb, Croatia; boris.brkljacic@mef.hr; 3 Department of Abdominal Surgery, University Hospital Split, University of Split School of Medicine, Spinčićeva 1, 21000 Split, Croatia; mboric@kbsplit.hr

**Keywords:** breast, breast cancer, MR diffusion, NME lesions, ADC

## Abstract

Objectives: This study aimed to investigate whether the apparent diffusion coefficient (ADC) maps values of breast lesions presenting as non-mass enhancement (NME) on MRI could predict benign or malignant pathohistological findings. Materials and Methods: This retrospective single-center study included 136 female patients with NME and corresponding ultrasound correlate and a subsequent ultrasound-guided core needle biopsy. The patients were subdivided into benign or malignant subgroups based on pathology reports, which served as the gold standard. Blinded to the pathological results, two radiologists independently measured the ADC values of the depicted NME using punctate, 10 mm and whole tumor regions of interest (ROIs) wherever applicable. The mean of all measurements was also analyzed and compared with the pathologic subdivision. Results: The sensitivity of whole tumor ROI in detecting benign NME is 91% compared to 74% for 10 mm ROI and 78% for punctate ROI. No significant differences in ADC values were observed when comparing fatty breast tissue and dense breast tissue. Conclusions: There were differences in ADC values between benign and malignant findings using all types of measurements, where the whole tumor ROI was the most sensitive.

## Simple Summary

Everyday use of breast MRI led to an increase in the number of visualized non-mass enhancement whose characterization is still a clinical problem. The aim of this retrospective study was to show whether the visualized non-mass enhancement can be more precisely characterized with the help of delineating non-mass enhancement on subtraction, where it is best seen, and transferring the marked section to the apparent diffusion coefficient map, where it can be quantified.

## 1. Introduction

Breast magnetic resonance imaging (MRI) is a useful diagnostic tool for detecting changes in the breast; however, there is room for improvement by accurately addressing specific pathological conditions [1,2,3,4]. Non-mass enhancement (NME) is a unique diagnostic challenge, covering a broad spectrum of alterations, ranging from benign, hormone-dependent parenchymal enhancement to invasive tumors, all of which may exhibit similar MR morphology.

Though functional diffusion sequences, such as diffusion-weighted imaging (DWI) with corresponding apparent diffusion coefficient (ADC) maps, have improved lesion characterization in masses, their effectiveness in analyzing NME remains inconsistent [1,5,6,7,8,9,10]. Notably, up to one-third of NME lesions lack a clear equivalent of DWI [7]. Moreover, the optimal approach for DWI/ADC analysis remains unclear, as different analysis methods yield varying results for the same lesions [10,11,12,13].

NME changes are best visualized on T1-weighted postcontrast subtracted images [7], where post-contrast images are subtracted from pre-contrast images to enhance changes in tissue vascularity, thereby enhancing the contrast between lesions and surrounding tissues.

Although the whole tumor ROI method has been well-established for analyzing mass lesions, a small number of studies analyzed the whole volume of NME. Furthermore, in these studies, most authors delineated the whole volume of NME directly on ADC maps [10]. Given that NME is best visualized from dynamic contrast-enhanced (DCE) subtracted images [8], this study adopted a slightly different approach that involved delineating the whole volume of NME on each slice visible on DCE subtracted images with a corresponding ADC correlate. Although this approach is not completely novel, as previously shown by Chen et al. in a published study on breast lesions in general, to the best of our knowledge, there are no similar studies that were focused solely on NME [14].

Moreover, studies that included NME had a few cases with solely NME. For instance, there were 12 cases in a paper from Chen et al. [14] and 4 from Partridge et al. [15]. Liu et al. [16] analyzed ADC values of NME but did not use the presented method.

Our study aimed to assess whether adopting a previously described measurement method could improve the accuracy of predicting the malignancy of NME lesions.

Additionally, given that several published reports showed differences in ADC values of fibroglandular tissue regarding breast densities [17], we pondered whether breast density could possibly affect the ADC values of NME. Accordingly, the aim of the study was also to analyze the correlation between the ADC values of benign and malignant lesions across two subgroups based on the density of the fibroglandular parenchyma.

## 2. Materials and Methods

### 2.1. Patients

This single-center study obtained approval from the Ethics Committee at our institution and was conducted in accordance with the Declaration of Helsinki. In this retrospective study, the requirement for informed consent was waived.

The local hospital database was retrospectively searched to identify patients who underwent multiparametric breast MRI between January 2020 and June 2023. The inclusion criteria were female patients older than 18 years who underwent an initial breast MRI examination, subsequently revealing the presence of NME categorized as BI-RADS 4 or 5. In accordance with the BI-RADS categorization, all patients underwent ultrasound-guided core needle biopsy. To be included in the study, MR-identified NME lesions needed to have a corresponding ultrasound (US) correlate. The obtained specimens underwent pathological examination, classifying patients into benign or malignant subgroups. Malignant cases included ductal carcinoma in situ, invasive ductal carcinoma, and invasive lobular carcinoma, while benign cases included fibroadenoma, adenosis, inflammation, intraductal papilloma, fat necrosis, and fibrocystic changes. Exclusion criteria involved patients with B3 lesions and individuals with poor-quality DWI correlates or inadequate subtraction images. Furthermore, as explained in the introductory section, two subgroups were formed based on breast density: Group A (ACR a and b) and Group B (ACR c and d).

**Figure 1:**
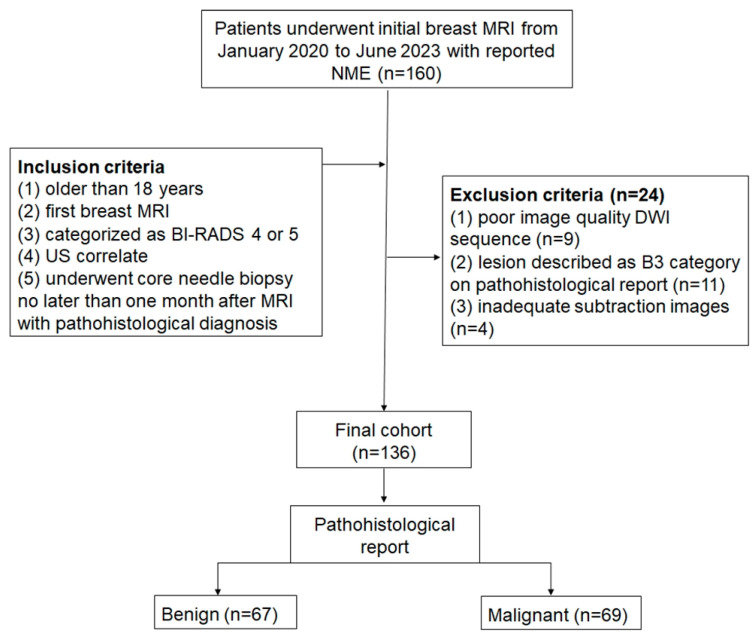
Flow chart of the patient enrollment procedure. Acronyms used: MRI, magnetic resonance imaging; NME, non-mass enhancement; BI-RADS, Breast Imaging-Reporting and Data System; US, ultrasound; DWI, diffusion-weighted imaging.

Based on prior reports, achieving 80% statistical power (α = 0.05 and β = 0.2) required a sample size of 70 patients for each group to detect a 10% difference in AUC [8]. Ultimately, 136 patients were included (Figure 1).

### 2.2. MR Scanning Protocol

Patients were scanned using 2 types of scanners: 1.5 T Aera and 3T Vida MRI scanners (Siemens Healthineers, Erlangen, Germany) because previous reports suggested no difference in absolute ADC values regarding the employment of different MR field strengths [10,18].

All patients underwent the same comprehensive breast MRI protocol, including intravenous administration of a gadolinium-based contrast agent.

The protocol performed using the 1.5 T MR scanner was as follows: (1) localizer, (2) axial T1-weighted images (TR/TE at 8.6/4.7 ms, FOV of 340 mm × 340 mm, and a slice thickness of 1.6 mm), (3) axial turbo spin echo inversion recovery sequence with magnitude reconstruction or TIRM (TR/TE/TI values of 5200/57/170 ms, FOV of 347 mm × 370 mm, and a slice thickness of 4 mm), (4) DWI sequence using special attenuated inversion recovery or SPAIR (TR/TE, 5000/73 ms, FOV 176 mm × 400 mm; thickness 4.5 mm), three b values of 50, 400 and 800 s/mm^2^, (5) axial T1 fat suppressed weighted images used for dynamic (TR/TE values of 5.3/2.4 ms, FOV of 375 mm × 300 mm, and a slice thickness of 1.6 mm), and (6) subtracted images generated automatically by the operating system.

The protocol performed using the 3T MR scanner: (1) localizer, (2) axial T1- weighted images (TR/TE values of 5.5/2.59 ms, FOV of 250 mm × 250 mm, and a slice thickness of 2 mm), (3) axial short tau inversion recovery sequence or STIR (TR/TE/TI values of 5270/66/170 ms, FOV of 347 mm × 370 mm, and a slice thickness of 4 mm), (4) DWI sequence using special attenuated inversion recovery or SPAIR (TR/TE values of 8900/58 ms, FOV of 176 mm × 350 mm, and a slice thickness of 3 mm), three b values of 50, 400 and 800 s/mm^2^, (5) axial T1 fat suppressed weighted images used for dynamic (TR/TE values of 4.6/1.69 ms, FOV of 360 mm × 300 mm, and a slice thickness of 2 mm), and (6) subtracted images generated automatically by the operating system.

In the imaging protocol, six subtraction images were initially acquired. The first image was captured at the two-minute mark, followed by additional images taken every subsequent minute. Due to the susceptibility of subtraction images to motion artifacts, motion correction was applied prior to analyzing them whenever possible or practical. The total acquisition time was approximately 15 min.

### 2.3. Image Evaluation and Histopathology

Two breast radiologists, each with at least five years of experience in breast MRI, analyzed the MRIs of the patients using a Syngo.via equipped with MR Breast Workflow (Siemens Healthineers, Forchheim, Germany) on the diagnostic monitor (1600 × 1200 pixel resolution) without knowing the existing histopathological report. Each radiologist reviewed the MRI images and recorded the distribution and internal enhancement of NME. Given the lack of standardization in measuring ADC values for NME, our readers employed three different measurement approaches. Consistent with previous studies [7,8,16], measurements were carried out using both punctate and 10 mm region of interest (ROI) methods. Avendano et al. [7] described acquiring a punctate ROI on the darkest part of the lesion on ADC maps. Selecting the area for the 10 mm ROI was also based on finding the darkest part of the lesion on the ADC maps, wherever possible. However, due to the several NME lesions presenting a linear distribution, obtaining a 10 mm ROI proved unattainable for every lesion.

**Figure 2:**
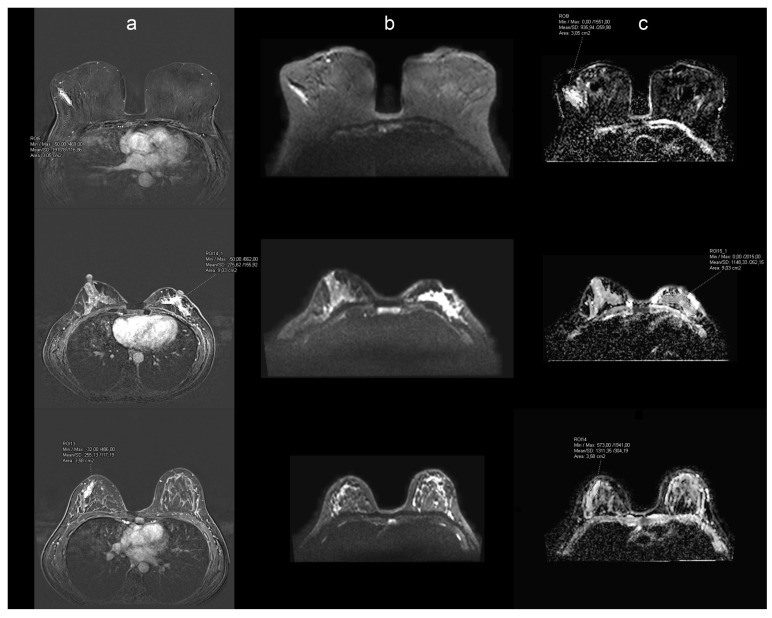
Example of MR workflow used for analysis. Each reader delineated non-mass enhancement (NME) on subtracted dynamic contrast-enhanced images (DCE) (column (**a**)), columns (**b**) and (**c**) presented a corresponding diffusion-weighted sequence at b 800 (**b**) and apparent diffusion coefficient (ADC) map (**c**). The delineated area on the DCE image was copied and transferred to the corresponding ADC map along with the calculated mean. The same was performed on each slice where NME was visible on DCE, and the mean of the whole volume ADC value was further calculated. The first row shows linear NME pathohistologically proven invasive ductal carcinoma, the second row corresponds to invasive lobular carcinoma presented as clumped NME, and the third row shows linear NME pathohistological characterized as periductal inflammatory changes without proof of malignancy.

Subsequently, the third method for measuring ADC value involved freehand delineation of the whole tumor ROI on subtraction images to achieve more precise delineation. During the delineation process, the readers correlated the DCE images to TIRM to exclude the cystic or necrotic areas. Similar approaches were used by Partridge et al. [15], primarily for mass lesions. The subtraction image used for delineation purposes was captured in the third minute of the protocol when most non-mass areas were readily visible. Due to variations in layer thickness between subtraction and diffusion images, measurements were conducted solely on the subtraction series that correlated with the diffusion series. Each delineated area was then transferred to the corresponding ADC map using the Syngo.via MR Breast Workflow (Siemens Healthineers, Erlangen, Germany), allowing accurate transfer of delineated volume to the same organ position (Figure 2).

Histopathological analyses of subsequent core needle biopsy specimens were examined by an experienced breast pathologist. Biopsied samples were immersed in neutral buffered formalin overnight and then embedded in paraffin. Immunohistochemical staining was performed on 4 μm sections according to standard pathohistological guidelines (ER, PR, and HER2 antibodies for malignant lesions) [19,20].

### 2.4. Statistical Analysis

Statistical analysis was performed using the MedCalc^®^ statistical software, version 22.019 (MedCalc Software Ltd., Ostend, Belgium; https://www.medcalc.org; accessed on 5 August 2023) with *p* < 0.05 considered statistically significant.

Inter-observer agreement was assessed using the intraclass correlation coefficient (ICC), supported by Bland–Altman plots. The ICC value, denoted by the r coefficient (Pearson’s correlation coefficient for a sample statistic), ranges from −1 to 1, where extremes indicate perfect linear negative or positive correlations.

Initial analyses of continuous variables and normality assessments were conducted using the Kolmogorov–Smirnov test. Because all variables were normally distributed, intergroup comparisons were performed using Student’s *t*-test. Further analyses included Pearson’s χ2 test and ROC curves analysis with the area under the curve (AUC). The AUC value ranges from 0 to 1, with 0 indicating the complete reciprocal of the classes, and 1 a perfect delineation between the two groups representing. For each delineation technique, the ADC threshold was chosen for acquiring the highest combination of sensitivity and specificity.

## 3. Results

### 3.1. Patients and Lesion Characteristics

A total of 136 female patients were included in the study (mean age = 53.7 ± 13.2 years). Of these, 67 patients (49.3%) were diagnosed with benign lesions, while 69 patients (50.7%) had malignant lesions. An analysis of the distribution and internal enhancement revealed statistically significant differences only in focal distribution, which were more prevalent in benign lesions (Table 1). No other statistically significant differences were observed.

### 3.2. Inter-Observer Agreement

In assessing the ADC values of NME between two readers, both the degree of consistency and absolute agreement among measurements had an ICC value of 0.85 (95% CI 0.78–0.9), indicating a high level of agreement. The coefficient of variation was calculated as 13.37% (95% CI 11.37–15.41).

**Figure 3:**
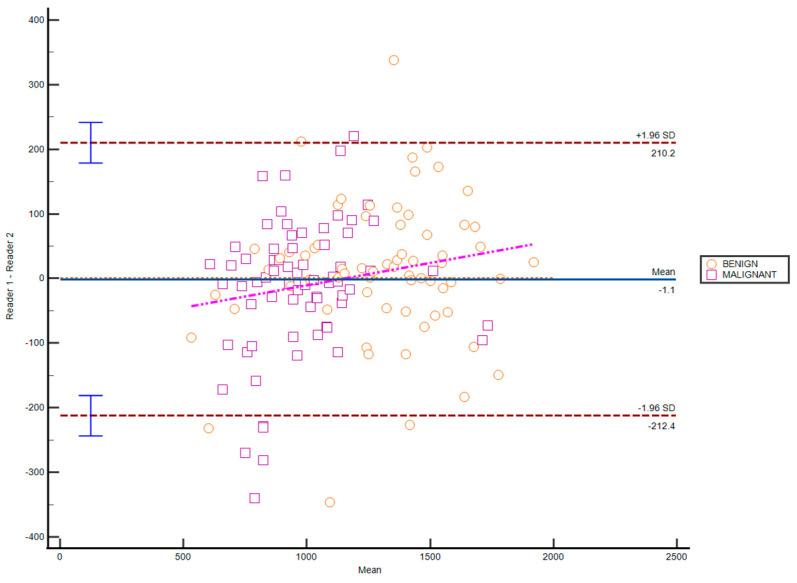
A Bland–Atman’s plot created to compare measurements between Reader 1 and Reader 2 against the mean of the two readers and a representation of the limits of agreement (dotted line), from −1.96 SD to +1.96 SD.

Bland–Atman’s analysis did not reveal a significant difference between the two readers, affirming the reliability of the measurements. The coefficient of repeatability is 327.71 (95% CI 287.55–381) (Figure 3).

### 3.3. Differentiation of Benign and Malignant Breast Lesions, Including Differences Related to Breast Composition Using Different Measurement Methods

After establishing a high level of agreement between the two readers, a comprehensive analysis was conducted. Further analyses used the mean values between two readers for punctate, 10 mm, and whole tumor ROI. Significant differences in ADC values were observed between malignant and benign lesions of all included patients among all three measurement methods (Table 2).

Further analyses were based on ROC curve analyses, including sensitivity, specificity, positive predictive value (PPV), negative predictive value (NPV), diagnostic accuracy, and the area under the curve (AUC).

**Figure 4:**
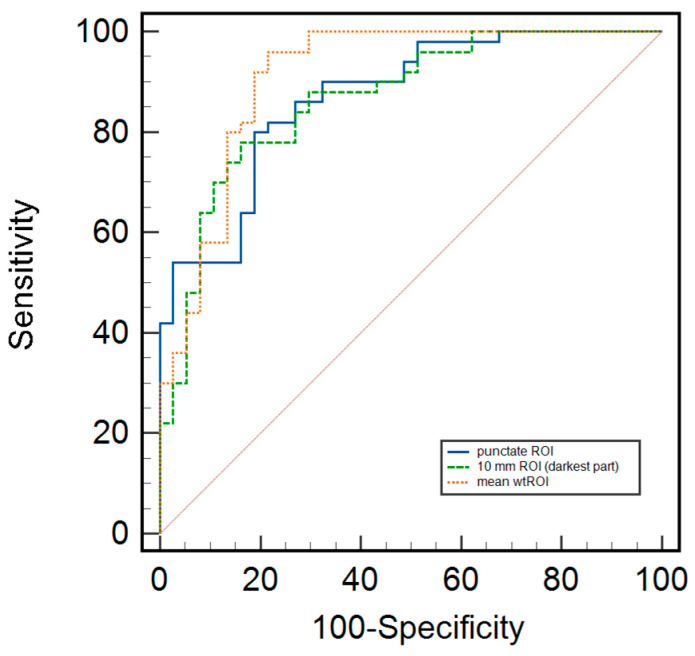
ROC curves for the ADC values of non-mass lesions for punctate, 10 mm, and whole tumor ROI.

The ADC thresholds for the different ROIs were as follows: the threshold for the punctate ROI was 0.810 × 10^−3^ mm^2^/s, with a sensitivity of 78%, specificity of 76%, PPV of 77%, NPV of 77%, diagnostic accuracy of 77%, and AUC of 0.772 (95% CI 0.688–0.837). For the 10 mm ROI, the threshold was 1.073 × 10^−3^ mm^2^/s, with a sensitivity of 74%, specificity of 86%, PPV of 88%, NPV of 71%, diagnostic accuracy of 79%, and AUC of 0.802 (95% CI 0.711–0.885). The whole tumor ROI threshold was 1.190 × 10^−3^ mm^2^/s, with a sensitivity of 91%, specificity of 64%, PPV of 72%, NPV of 88%, diagnostic accuracy of 78%, and AUC of 0.775 (95% CI 0.722–0.863) (Figure 4). In the current setting, for whole tumor ROI, the rule-out criterion for excluding malignancy was an ADC value of 1.505 × 10^−3^ mm^2^/s with 97.1% sensitivity (95% CI 89.9–99.6).

According to breast composition, all patients were divided into two subgroups for further analysis (Table 3). There were no differences in the incidence of malignant as opposed to benign lesions among both groups.

Given previous findings confirming differences in ADC values between benign and malignant lesions, further sub-analysis related to breast composition and different measurement methods was conducted (Table 2).

Based on all three measuring methods, significant differences in ADC values between malignant and benign cases were found in both groups in terms of breast density.

## 4. Discussion

In this study, we analyzed whether different approaches to ADC measurement might improve diagnostic accuracy in distinguishing between benign and malignant breast lesions presented as NME. Additionally, building upon previous studies [15,18], we examined how breast composition affects the differentiation between benign and malignant lesions using the analysis approach proposed in this study.

Based on the analyzed assessment methods we observed different thresholds for each method, which was expected due to varying ROI sizes. However, the 10 mm ROI could be used only where there was enough lesion area available. As a result, identifying the darkest part was sometimes challenging, leading to a calculated threshold that was slightly higher than that of the whole tumor ROI. Moreover, whole tumor ROI corresponding to subtracted DCE images appeared to be the most sensitive in distinguishing between benign and malignant lesions. This approach was used by Partridge in their study, primarily for mass lesions [15]. We observed a sensitivity of 91% and a specificity of 64%, representing a 13% improvement in sensitivity compared with punctate measurements and 17% for 10 mm ROI measurements. Consistent with previous reports [5], malignant lesions exhibited lower average ADC values compared with benign ones using a threshold of 1.190 × 10^−3^ mm^2^/s. Analyzing DWI and corresponding ADC in NME lesions, which lack classical volume, poses challenges. The suggested method of delineating the region with NME on each slice visible in DCE subtracted images and transferring that delineated area onto the anatomically corresponding ADC map seemed reasonable. Additionally, the high inter-reader reproducibility of measurements (ICC 0.78–0.9) suggested that this approach could be reliably implemented in clinical practice. Moreover, using a high sensitivity threshold of 1.505 × 10^−3^ mm^2^/s, we could exclude malignancy with a sensitivity of 97% using whole tumor ROI.

A similar approach was employed in our previous study on colonic cancer, where we demonstrated that whole tumor measurement for obtaining ADC values served as an additional tool for predicting tumor grade and nodal infiltration [21]. Several studies have also used DCE and DWI/ADC correlations for breast lesion characterization; however, none had exclusively investigated NME lesions [14,22,23].

Our results align with previous findings regarding the other two analysis methods—punctate and 10 mm ROI [24,25,26,27,28,29]. However, the study by Avendano et al. [7] observed that ADC mapping in NME lesions had limited diagnostic accuracy regardless of the approach to measuring an ROI. In contrast, in this paper, we employed a different approach in whole tumor measurement, yielding different results.

Previous proposals suggested that the amount of fibroglandular tissue and its related tissue cellularity might influence diffusibility and ADC values [1]. Additionally, as Partridge et al. showed, due to intravoxel contribution from a fat signal, the amount of fibroglandular tissue could result in underestimated ADC values for both tumor and normal tissue [15]. Therefore, we conducted a sub-analysis related to breast tissue composition, confirming that there were differences in ADC values between malignant and benign lesions, independent of the amount of fibroglandular tissue.

Several limitations should be acknowledged. Firstly, our inclusion criteria were limited to patients with ultrasound correlates, as our biopsy specimens were obtained under ultrasound guidance. Ideally, biopsies should be performed under MR guidance. However, due to the number of patients included and our clinical setting, this was not feasible. Additionally, the retrospective nature of the study and its lack of use for decision-making might introduce selection bias, as only patients with BI-RADS 4 and 5 lesions were included.

Finally, there was a limitation in using the whole volume approach in transposing the delineated part of NME from the subtracted T1-DCE figure to the ADC map because there was no perfect geometrical overlap between images. However, MRs that had visually evident low shim quality were excluded, as stated in the Methods. This approach was not completely novel, given that other authors have already used similar methods in different settings [15,21].

## 5. Conclusions

The ADC value obtained from the whole volume NME lesion measurement and correlating with DCE images could serve as an additional factor in the decision-making process for distinguishing between benign and malignant lesions. This should be further investigated in larger studies.

## Figures and Tables

**Table 1:** NME ^a^ lesions and MR imaging descriptors.

	Malignant (n = 69) (%)	Benign (n = 67) (%)	*p*-Value
**Distribution**			
Focal	21 (31)	35 (52)	**0.049**
Linear	9 (13)	13 (19)	0.37
Segmental	12 (17)	4 (6)	0.06
Regional	16 (23)	8 (12)	0.12
Multiple regions	7 (10)	2 (3)	0.12
Diffuse	4 (6)	5 (8)	0.72
**Internal enhancement patterns**			
Homogeneous	6 (9)	8 (12)	0.57
Heterogeneous	47 (68)	55 (82)	0.35
Clumped	10 (14)	3 (5)	0.06
Clustered-ring	6 (9)	1 (1)	0.08

^a^ NME—non-mass enhancement.

**Table 2:** ADC ^a^ values of the benign and malignant lesion using punctate, 10 mm, and whole tumor ROIs were subgrouped into all included patients, Groups A and B. Group A included ACR a and b from the BI-RADS lexicon, while Group B comprised ACR c and d.

ADC Value (×10^−3^ mm^2^/s)	Mean ± SD		*p*-Value
	Malignant	Benign	
**Punctate ROI ^b^**			
All included	0.618 ± 0.282	0.985 ± 0.311	**<0.01**
Group A	0.526 ± 0.235	0.826 ± 0.316	<0.01
Group B	0.687 ± 0.292	1.081 ± 0.271	<0.01
**10 mm ROI**			
All included	0.928 ± 0.237	1.357 ± 0.418	**<0.01**
Group A	0.868 ± 0.232	1.425 ± 0.702	0.03
Group B	0.984 ± 0.234	1.323 ± 0.259	<0.01
**Whole tumor ROI**			
All included	0.985 ± 0.213	1.294 ± 0.297	**<0.01**
Group A	0.912 ± 0.156	1.140 ± 0.342	0.004
Group B	1.038 ± 0.234	1.385 ± 0.225	<0.01

^a^ ADC—apparent diffusion coefficient, ^b^ ROI—region of interest.

**Table 3:** Breast composition is described as Group A, which included ACR ^a^ a and b from BI-RADS ^b^ lexicon, and Group B, which included ACR c and d.

	Malignant (n = 69) (%)	Benign (n = 67) (%)	*p* Value
Breast composition			
Group A (ACR a and b)	29 (42)	25 (37)	0.67
Group B (ACR c and d)	40 (58)	42 (63)	0.73

^a^ ACR—American College of Radiology, ^b^ BI-RADS—Breast Imaging-Reporting and Data System.

## Data Availability

Data are available upon request due to restrictions (privacy reasons).
